# Surgical techniques and strategies for the treatment of primary liver tumours: hepatocellular and cholangiocellular carcinoma

**DOI:** 10.1007/s10353-018-0537-x

**Published:** 2018-05-17

**Authors:** Eva Braunwarth, Stefan Stättner, Margot Fodor, Benno Cardini, Thomas Resch, Rupert Oberhuber, Daniel Putzer, Reto Bale, Manuel Maglione, Christian Margreiter, Stefan Schneeberger, Dietmar Öfner, Florian Primavesi

**Affiliations:** 1 Department of Visceral, Transplantation and Thoracic Surgery, Medical University of Innsbruck, Anichstraße 35, 6020 Innsbruck, Austria; 2 Department of Radiology, Medical University of Innsbruck, Anichstraße 35, 6020 Innsbruck, Austria

**Keywords:** Surgery, Hepatic resection, Primary liver tumours, Hepatocellular carcinoma, Cholangiocarcinoma

## Abstract

**Background:**

Owing to remarkable improvements of surgical techniques and associated specialities, liver surgery has become the standard of care for hepatocellular carcinoma and cholangiocarcinoma. Although applied with much greater safety, hepatic resections for primary liver tumours remain challenging and need to be integrated in a complex multidisciplinary treatment approach.

**Methods:**

This literature review gives an update on the recent developments regarding basics of open and laparoscopic liver surgery and surgical strategies for primary liver tumours.

**Results:**

Single-centre reports and multicentre registries mainly from Asia and Europe dominate the surgical literature on primary liver tumours, but the numbers of randomized trials are slowly increasing. Perioperative outcomes of open liver surgery for hepatocellular and cholangiocellular carcinoma have vastly improved over the last decades, accompanied by some progress in terms of oncological outcome. The laparoscopic approach is increasingly being applied in many centres, even for patients with underlying liver disease, and may result in decreased morbidity. Liver transplantation represents a cornerstone in the treatment of early hepatocellular carcinoma and is indispensable to achieve long-term survival. In contrast, resection remains the gold standard for cholangiocarcinoma in most countries, but interventional techniques are on the rise.

**Conclusion:**

Liver surgery for primary tumours is complex, with a need for high expertise in a multidisciplinary team to achieve acceptable outcomes. Technical developments and clinical stratification tools have optimized individual care, but further improvements in oncological survival will likely require enhanced pre- and postoperative systemic and local treatment options.

## Main novel aspects

- A short summary on the latest advances in open and laparoscopic liver surgery
- Update on recent developments to integrate surgery in the multidisciplinary treatment of primary liver tumours
- Review of recently published papers through literature search regarding surgical treatment of hepatocellular carcinoma and cholangiocarcinoma

## Introduction

Liver resections are performed to manage both benign and malignant pathologies, with the majority undertaken for primary or secondary liver tumours. These procedures have long been regarded as one of the most challenging in general surgery, due to high perioperative morbidity and mortality. With refinements of surgical techniques leading to reduced blood loss, enhanced rates of complete tumour resection and increased residual liver function, as well as better imaging, appropriate patient selection and modern anaesthesia [1], liver surgery has experienced great improvements in terms of postoperative outcome and long-term survival in the past decades [2].

This article focuses on technical aspects, strategies and outcomes of commonly applied techniques for the surgery of primary liver tumours, namely hepatocellular carcinoma and cholangiocarcinoma, indications that still remain most demanding within the field of hepatobiliary surgery. Topics covered include open surgical procedures, laparoscopic liver resection (LLR) and the role of systemic therapies within curative treatment concepts. Ablation and other local therapies will be discussed in the article on interventional oncology by Putzer et al. in this special edition of *European Surgery*.

## Techniques in liver surgery

### Anatomical vs. non-anatomical resection

Liver malignancies can either be excised by anatomical or non-anatomical resection. Anatomical resection (AR) is based on the understanding of the segmental anatomy of the liver, which in turn is based on the organ’s blood supply via the hepatic artery and portal vein, its drainage via the hepatic veins and the biliary drainage [3]. AR theoretically carries the advantage of less bleeding, as it avoids intrahepatic impairment of major vessels and also reduces the likelihood of leaving ischaemic liver tissue behind, since the blood supply to the remnant is preserved. Non-anatomical resections (NAR) are widely used for peripheral or superficial lesions, when the lesion crosses the boundary of multiple segments or in situations where the preservation of liver substance is of paramount importance. Particularly in cases of intrahepatic recurrent disease, which occurs in up to 50% of resections for liver malignancies, NAR are often used because of their greater parenchymal-sparing potential [4].

### Critical factors for planning hepatic resections

The following factors determine the planning of a hepatic resection:

- Location, distribution and number of tumour lesions
- Involvement of vascular and biliary structures
- Amount and quality of functional parenchyma, in- and outflow vascular supply and biliary drainage of the remaining liver after hepatectomy (the so-called future liver remnant, FLR)
- Comorbidities and fitness of the patient

Defining the transection plane implies evaluation of the relationship between tumour, major intrahepatic vessels and bile duct pedicles through preoperative imaging, mostly with a contrast-enhanced, multidetector computed tomography (CE-MDCT) scan. Further techniques include magnetic resonance imaging (MRI), which is especially beneficial to detect smaller additional intrahepatic lesions and the extent of tumour along the main bile ducts through magnetic resonance cholangiopancreatography (MRCP), positron-emission tomography (PET), contrast-enhanced ultrasound (CEUS) and endoscopic techniques including endoscopic retrograde cholangiopancreatography (ERCP) and endoscopic ultrasound (EUS).

Also, intraoperative ultrasound (IOUS) is routinely used during surgery, e. g. to assess the extent of tumour involvement of vessels such as the middle hepatic vein and to determine whether an extended resection is needed for a tumour-free margin [5].

Different to secondary liver tumours, a minimum of 1 cm tumour-free margin is recommended for primary liver tumours. Especially for hepatocellular carcinoma (HCC), anatomic resections aiming at 2 cm margins provide better survival outcome than narrow resection margins <1 cm and are advisable whenever an appropriate FLR volume and function is ensured [6].

### Strategies for manipulating liver volume

Several studies showed that the FLR needs a certain threshold of volume to be preserved to sustain metabolic, synthetic and detoxifying functions. While the removal of up to 75% of the total liver volume is feasible in young “healthy” patients, resection must be more conservative in the presence of underlying pre-existing liver disease (steatosis, fibrosis, cirrhosis) or in elderly patients. An approved strategy to manipulate the liver volume is selective occlusion of the portal branch, which causes atrophy of the ipsilateral and hypertrophy of the contralateral liver lobe [7]. Selective interruption of the portal flow can be achieved by either percutaneous portal vein embolization (PVE) or (less commonly) through surgical ligation. PVE is mostly used preoperatively for a right or extended right hemihepatectomy in cases when the FLR would otherwise be too small (usually <30% or less). Surgical ligation has been integrated together with tumour clearance of the FLR into a strategy for two-stage hepatectomy for initially unresectable, multiple bilobar liver tumours [8]. A further advancement of this strategy, the “associating liver partition and portal vein ligation for staged hepatectomy (ALPPS)”, was developed in 2010. By also splitting (parts of) the liver parenchyma along the planned resection line during the first step while preserving the arterial supply, a rapid and extensive growth of the FLR can be achieved to expand the rate of curative resection in selected subgroups of patients [9]. Resection is usually completed after an interval of around 10 days, when adequate hypertrophy of the FLR is documented [10]. Initially reported high rates of postoperative mortality and morbidity have clearly improved with increased experience and refined patient selection during the last few years; however, there is an ongoing discussion about the long-term oncological outcomes [11, 12].

### Preoperative patient selection

Despite technical advances, estimation of individual patient risk is crucial for stratification of patients to treatment options with different grades of invasiveness. A number of scores and tests are commonly used preoperatively to estimate perioperative risk, including the American Society of Anaesthesiology (ASA) grade, the Charlson Comorbidity Index (CCI) and Cardiopulmonary Exercise Tests (CPET). Each of the three has been shown to be predictive for postoperative outcome [13]. However, the potential risk faced by patients undergoing liver surgery always remains a sum of factors, such as comorbidity, physical fitness, the presence of coexisting liver diseases and the extent of liver resection.

### Techniques of parenchymal transection

Alongside the rapid increase of hepatic resections performed worldwide over the last decades, a broad spectrum of different surgical devices and techniques have been developed to facilitate the different aspects of hepatic surgery like haemostasis, vascular control and parenchymal transection.

The finger-fracture technique (digitoclasia) or clamp-crushing method (kellyclasia) are the most basic techniques for parenchymal transection, accompanied by the use of diathermia, clips or suture ligatures to secure vessels and bile ducts [14]. Unipolar or bipolar cautery as well as argon beamer coagulation is used for haemostasis during and after transection [15]. Nowadays, in many centres, ultrasonic dissection using the Cavitron Ultrasonic Surgical Aspirator (CUSA®, Valleylab Boulder, CO, USA) has become the standard technique for transection, allowing for meticulous division of the liver parenchyma while exposing vessels and bile ducts for selective ligature and haemostasis. Compared to conventional methods, the time for transection using CUSA® is usually longer. Although it was initially shown that CUSA® reduces blood loss, morbidity and mortality, a randomized controlled trial could not prove any benefit in terms of blood loss in liver resection when CUSA® was compared to clamp crushing [14, 16]. The water-jet is another dissection device, washing off softer liver tissue from vessels by using a high-pressure water jet. A randomized controlled trial by Rau et al. showed reduced transfusion requirements, decreased need for intermittent liver ischaemic time (Pringle manoeuvre) and shorter transection time compared to the CUSA® technique [17].

Additionally, numerous vessel-sealing devices like the Ligasure system® (Medtronic, USA) or the HARMONIC Scalpel® (Johnson & Johnson, USA) are often used in laparoscopic or non-anatomical resections for parenchymal transection to seal small blood vessels and bile ducts (up to 7 mm with recent generation devices) [14, 18].

A randomized controlled trial published in 2005 compared four different transection methods. Analysing resection time, blood loss, blood transfusion frequency and cost efficiency, the clamp-crushing methods remained the most efficient device compared to CUSA®, water-jet and dissecting sealer [19]. Recently, stapler hepatectomy has been proposed for parenchymal transection, leading to significantly shorter total duration of surgery compared to clamp-crushing or CUSA® resection in two randomized controlled trials. While this was accompanied by a diminished inflammatory response, there was no significant difference in terms of total blood loss or postoperative morbidity. Furthermore, stapler hepatectomy is usually only feasible in standard procedures with linear resection planes [20, 21].

## Hepatocellular carcinoma

### Epidemiology and pathogenesis

Hepatocellular carcinoma (HCC) is the fifth most common cancer worldwide and has risen to become the third most common cause of cancer-related deaths worldwide, accounting for over >800,000 deaths/year [22]. The incidence in Asian countries is up to ten times higher compared to the Western World countries due to the endemic presence of hepatitis B virus [23]. About 80% of HCCs develop on the background of alcohol-toxic or primary biliary cirrhosis. Other risk factors include fibrosis, steatosis, obesity, diabetes, aflatoxin B_1_ and several genetic metabolic diseases (hemochromatosis, Wilson’s disease, alpha1-AT-deficiency, glycogen storage diseases, etc.). The most commonly used tumour marker alpha-feto protein (AFP) has a rather low sensitivity and specificity of only 40–65% and 76–96%, subject to the cut-off value and the presence of viral hepatitis [24].

### Classification and surgical management options

**Fig. 1:**
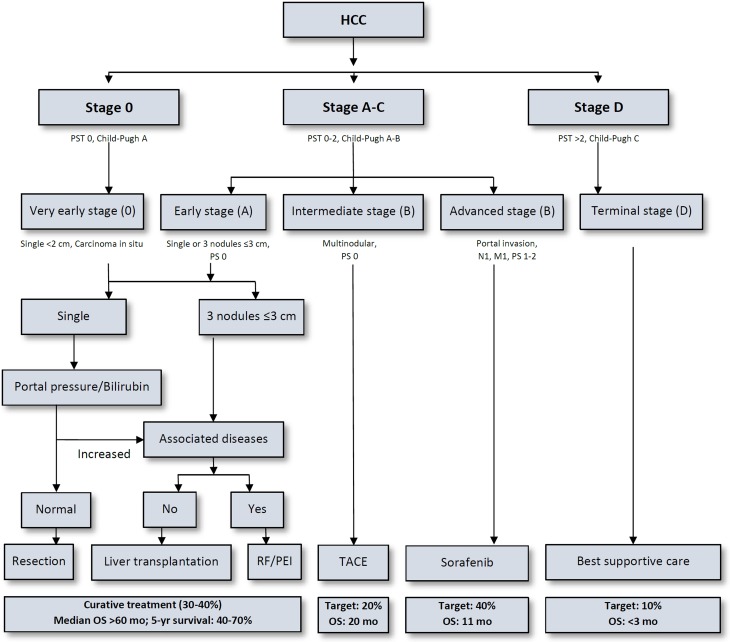
The BCLC Criteria for HCC Management; *cm* centimeter, *m1* metastasis present, *mo* months, *n1* nodal positive, *OS* overall survival, *PEI* percutaneous ethanol injection, *PST/PS* performance status, *RF* radiofrequency ablation, *TACE* transcatheder arterial chemoembolisation. (Adapted from [118])

Surgical management of HCCs around the world widely varies depending on availability of donor organs and local resources as well as expertise of centres. Selecting the appropriate treatment for these patients is challenging, with a need for individual decision-making especially because most HCC patients present with underlying liver disease (cirrhosis, steatosis, portal hypertension), while spontaneous HCC in a healthy liver is considered rare (<10%) [25]. Since patients with fibrosis or cirrhosis are at risk of decompensation following liver resection, these factors are incorporated in most staging systems to stratify treatment. Among several available staging systems intended for guidance in management of HCC patients, the Barcelona Clinic Liver Cancer (BCLC) staging system is probably the most frequently used worldwide (Fig. 1; [26]).

Although there is an increasing debate in the transplantation community about the suitability of the BCLC criteria in modern HCC therapies, especially for intermediate and advanced stages [27], the BCLC staging system gives a valid impression of the mainstays of decision-making in HCC patients: the grade of cirrhosis, performance status of the patient, number and size of the lesions as well as macrovascular invasion remain the crucial factors in choosing therapeutic options. Patients in BCLC stage 0 or A, eligible for potentially curative treatment (resection, orthotopic liver transplantation-OLT, or ablation) exhibit markedly improved prognosis. On the other side of the spectrum, patients with advanced (BCLC stage C) or terminal (stage D) disease most commonly portend a dismal prognosis of less than 12 months overall survival. It is well known that the tumour size correlates with micro- and macrovascular invasion. The presence of any macrovascular invasion is a major risk factor for poor survival in HCC patients and should therefore always be evaluated by preoperative imaging [28, 29].

Concerning early and intermediate-stage disease, several criteria for indication of OLT are used in centres worldwide, mostly the Milan criteria (single tumour ≤5 cm or maximum three tumours ≤3 cm, no vascular invasion or metastasis) [30]. However, there is increasing evidence that the strict application of the Milan criteria might deprive patients from potentially beneficial OLT. Therefore, extended criteria were developed, such as the University of California San Francisco (UCSF) criteria (1 lesion ≤6.5 cm or 2–3 lesion each ≤4.5 cm with a total diameter of ≤8 cm) [31] or the new Milan “up-to-seven” criteria (sum of the size of the largest tumour and the number of tumours below seven and no macrovascular invasion) [32].

Further markers for poor tumour biology and high post-OLT recurrence rates are: no response to bridging treatment, initial high AFP and positive enhancement on PET-CT. A combination of PET-CT-positivity and AFP >200 ng/ml could predict tumour recurrence better than the Milan criteria in a recent living donor transplantation study [33].

Therefore, even patients with BCLC intermediate stage may proceed to liver transplant [27]. Local or systemic therapy should be given for all HCCs whenever possible [34, 35]. After a recurrence-free follow-up of 3–6 months, OLT should be considered especially for patients without high-risk factors.

Comparing resection and OLT, a recent meta-analysis of observational studies suggested superior overall survival (OS) and disease-free survival (DFS) rates after OLT for patients with Child A cirrhosis and within the Milan criteria. Since randomized trials are still missing [36] and due to shortage of donor organs, surgical resection of HCCs in selected patients still comprises a valid option as a definitive treatment or as a bridge to transplantation. The multidisciplinary process for selection of patients eligible for minor or major resection has been described by several high-volume centres, e. g. the University of Texas MD Anderson Cancer Centre (Houston, USA) as depicted in Table 1; [37].

**Table 1:** MDACC Criteria for Resection in Chronic Liver Disease. (After [37])

Minor Resection	Major Resection
Child–Pugh A Normal liver function tests (Bilirubin ≤1.0 mg%) Absence of ascites Platelets > 100,000/µl	Criteria for minor resection + Absence of portal hypertension Portal vein embolisation (PVE) for FLR <40%

Furthermore, the extent of resection (enucleation, segmentectomy, bi- or trisectionectomy) may also be guided by the use of functional liver tests such as indocyanine green clearance (ICG clearance) or other markers recently investigated for prediction of post-hepatectomy liver failure (PHLF) such as von Willebrand Factor [38–40]. In cases of insufficient size of the FLR (<40%), PVE might decrease the risk of PHLF, although resection may be challenging due to possible severe adhesions on the diaphragm and inflammatory reaction around the hilum. On the other hand, PVE (in selected cases combined with sequential arterial embolization) might even improve long-term survival in HCC patients [41]. Regarding application of the ALPPS procedure for HCC, data are limited in the multicentre international ALPPS registry, contrary to the situation with secondary liver tumours or cholangiocarcinoma. However, there are series from Hong Kong [42] and Italian groups [43] demonstrating that this technique is feasible in cirrhotic livers with acceptable postoperative morbidity and mortality, although fibrotic liver tissue shows less rapid volume growth compared to healthy parenchyma. Parallel to the advances for safer surgery in the whole ALPPS cohort [12], refinement of strategic details such as partial parenchymal splitting, laparoscopic stage-1 surgery, extension of the interstage interval to a median of 15 days or testing the interval FLR function might further enhance applicability and safety in HCC patients, too.

In general, ALPPS seems reasonable for the following HCC patients [10]:

1. Child A cirrhosis, no portal hypertension (young patients) or non-cirrhotic liver,
2. Conventional two-stage approach not feasible due to PV branch invasion,
3. Previous PV embolization technically failed.

Regarding the debate of AR versus NAR in HCC, two meta-analyses have been published in the past years [44, 45]. In summary, AR shows improved outcomes compared to NAR concerning DFS and OS, but results are conflicting due to more cirrhotic patients in the NAR group and should therefore be interpreted with caution. Furthermore, a retrospective single-centre analysis of patients with preserved liver function from Japan did not show any difference between these two groups of patients [46].

Contrarily to cirrhotic patients, spontaneous HCC without underlying liver disease shows a different characterisation. These tumours usually present in a late stage, with large diameter and frequent pulmonary metastasis during follow-up. Vascular involvement is not generally considered a contraindication and more aggressive indications for resection instead of transplantation might be reasonable, even with the use of intraoperative bypass for large tumours. Fibrolamellar HCC is a less common subtype of HCCs arising in non-cirrhotic livers, with a very different tumour biology and frequent recurrence. Resection of these tumours results in 5‑year OS rates of up to 65% compared to 50% after OLT [47].

### Diagnostics pre- and post-treatment

Most HCCs are asymptomatic and either diagnosed as an incidental finding or through routine surveillance of high-risk populations (e. g. patients with cirrhosis) by abdominal sonography combined with measurement of serum AFP [24].

Further assessment usually comprises MD-CT and MRI with hepatocyte-specific contrast enhancement (e. g. gadoxetic acid, GDA). Typically, HCCs show hypervascular enhancement with characteristic early contrast uptake and portal venous washout with a delayed enhancing outer rim “capsule”. Comparing CT and MRI for evaluating HCC, both have advantages and disadvantages. While CT is more sensitive to detect invasion of hepatic vascular structures, GDA liver MRI with diffusion-weighted imaging (DWI) is especially useful for detecting dysplastic nodules and HCC lesions of smaller size (1–2 cm). Recently, the 2017 update [48] of HCC LI-RADS (liver imaging reporting and data system) criteria was presented, clearly emphasizing the value of structured radiological reports. Applicability of MRI might be limited in patients with ascites, claustrophobia or restricted ability to hold breath for 15 s.

### Evaluating portal hypertension before surgery

As previously stated, the BCLC criteria are considered outdated by some authors for subgroups of patients, since there are good data for curative-intent treatment even in stage B (multinodular) and C (macrovascular invasion). Also, although a well-known risk factor for PHLF and impaired long-term outcome, portal hypertension itself is not anymore considered a strict contraindication for resection. Data from Asia for example show respectable 5‑year OS rates of 56% after HCC resection in the presence of PH compared to 71% in the non-PH group [49]. For evaluation of PH, assessment of the hepatic venous pressure gradient (HVPG, i. e. the difference between the wedged and the hepatic venous pressure) is the gold standard. However, although with a very low morbidity, HVPG measurement is an invasive method and is also not applicable in biliary type cirrhosis (e. g. due to CCC/HCC mixed type tumours). There is supporting evidence that non-invasive evaluation of liver fibrosis through liver stiffness measurement by transient elastography (Fibroscan®, Echosens, France) is an at least equivalent predictor for postoperative outcome [50, 51]. Finally, a history of clinical signs for complicated PH (ascites, encephalopathy or varicose bleeding) entails markedly increased postoperative complications and is therefore still a contraindication even in specialised liver centres [52].

### The role of laparoscopic surgery in HCC

Despite the vast experience with laparoscopy in hepatobiliary surgery [53], there is still a significant learning curve for LLR [54], wherefore the complexity of different laparoscopic procedures has been risk stratified by scoring systems [55] and a stepwise manner to introduce this approach in individual centres is advisable. Tumours favourable for LLR are typically solid lesions <5 cm located at segments II, III, IVb, V or VI, not close to major vascular trunks and without need for vascular and biliary reconstruction. Expertise is especially important in cirrhotic patients, with a substantial risk of bleeding and increased difficulty of mobilisation and identification of crucial structures. On the other hand, major benefits of laparoscopy in cirrhotic patients are reduced postoperative ascites, a lower rate of PHLF [56] and reduced blood loss with less need for transfusion in experienced hands. Also, laparoscopy decreases pulmonary complications in patients with major liver resection [57]. Altogether, this results in a reduced length of stay compared to open surgery. While there are still no randomized trials comparing laparoscopic versus open resections, three single-centre studies with propensity score-matched analysis showed comparable to favourable perioperative short-term outcomes and provided some evidence for possible long-term survival benefits after laparoscopic surgery for HCC [58–60]. As a consequence, within the last few years, LLR for HCC has become the standard practice in many centres at least for minor resections.

### Adjuvant treatment after curative treatment

Currently, guidelines do not routinely recommend adjuvant systemic treatment after resection or transplantation for HCC [61]. The positive results with palliative sorafenib reported for advanced HCCs raised hope for a similar effect in the adjuvant setting, but a recently published randomized, large-scale, international multicentre study (STORM trial) showed no difference in terms of DFS or OS compared to placebo [62]. When resection is applied in intermediate- or advanced stage patients, adjuvant sorafenib has shown some benefit in retrospective case-controlled studies [63]. Meta-analysis of RCTs of adjuvant interferon after resection showed a positive effect, but almost all published studies have been conducted in Asian patients and this could not be confirmed in Western cohorts [61]. In HCCs with vascular invasion or other high-risk factors such as poor differentiation and tumour diameter >5 cm, adjuvant transcatheter arterial chemoembolization (TACE) may provide better control of recurrence risk, especially with multiple treatment sessions [64, 65].

## Cholangiocarcinoma

Cholangiocarcinoma (CC) is a very heterogenous and rare group of neoplasms originating from the intrahepatic or extrahepatic (perihilar or distal) bile duct epithelium. This section will focus on intrahepatic (ICC) and hilar extrahepatic CC (hECC), since distal tumours are usually treated with bile duct and/or pancreatic resection alone and mostly do not require liver surgery.

### Classification

According to their anatomic relationship to the liver, CCs are either classified as intrahepatic or (perihilar or distal) extrahepatic CCs (ECC) [66–69]. Up to 5–20% of all CCs are intrahepatic and arise from peripheral bile ducts within the liver parenchyma proximal to the secondary biliary subdivisions [66]. Within the ECC, the majority (60–70%) account for hECC (termed “Klatskin” tumours) involving the confluence of the right and left main hepatic ducts [69]. Distal extrahepatic neoplasms represent up to 20% and 5% are multifocal. Hilar carcinomas can be subdivided according to the Bismuth classification [66, 69]:

- Type I: below confluence of left and right main hepatic ducts.
- Type II: reaching the confluence but not involving left or right main hepatic ducts.
- Type III: extending from the confluence to either the right (IIIa) or left (IIIb) main hepatic duct.
- Type IV: multicentric or bilateral intrahepatic segmental involvement; or involving the confluence and both right and left hepatic ducts.

Besides the anatomical classifications, tumours can be categorized according to their morphological appearance in mass-forming, periductal- or intraductal-infiltrating. The intraductal type carries the best prognosis while the periductal type is associated with the worst outcome [70]. Histopathologically, adenocarcinoma is the most frequent type, accounting for 90% of cases, whereas other histological variants, including papillary or intestinal type adenocarcinoma, adenosquamous carcinoma, squamous cell carcinoma and oat cell carcinoma, each comprise <5% of cases [71].

### Epidemiology

The epidemiology of CC and its subtypes displays enormous geographic differences reflecting the distribution of different risk factors, both environmental and genetic alike [72]. CC is the second commonest primary hepatic tumour worldwide after HCC [73, 74].

CC accounts for nearly 3% of all gastrointestinal cancers and 10–15% of liver malignancies diagnosed worldwide [74, 75]. Incidence and mortality rates for ICC have risen steeply and steadily across the world over the past few decades with concomitant falls in ECC rates [66, 73–82].

CC affects middle-aged and elderly individuals, the average age at time of presentation worldwide is 50 years. Aging is directly associated with a progressive increase in incidence, with the risk being slightly higher in men than women (male to female ratio 1.5:1) [66, 75, 83, 84].

### Risk factors

Over the past decades, several risk factors for CC associated with chronic inflammation of the biliary epithelium have been identified, although only accounting for less than 30% of all CC cases [85–88]. The most predisposing factor for CC is primary sclerosing cholangitis (PSC), with an estimated cumulative lifetime risk of 5–40% and a median interval between diagnosis of PSC and occurrence of CC of 2–2.5 years [75, 87, 89]. Other known risk factors for both ICC and ECC are age >65 years and liver flukes (*Opisthorcis viverrini* and *Clonorchis sinensis*). Risk factors especially associated with ICC are hepatitis-c virus (HCV) infection, hepatitis-b virus (HBV) infection and hepatolithiasis [72, 88, 89].

### Clinical presentation

The clinical presentation of cholangiocarcinoma depends on the location of the tumour. While ICC are usually asymptomatic or show unspecific symptoms, hECC usually present with features of biliary obstruction such as jaundice, pale stool, dark urine and pruritus. Cholangitis is uncommon without priory biliary endoscopy. The majority of patients are diagnosed with locally advanced or metastatic disease, causing weight loss and abdominal pain [66].

### Initial evaluation

Preoperative evaluation aims to exclude benign causes of biliary obstruction, to identify patients with early stage tumours who may benefit from surgery and to provide palliative biliary stenting and systematic treatment to those with advanced tumours and preserved performance status. According to the guidelines of the British Society of Gastroenterology, patients with suspected cholangiocarcinoma should at least receive a combined MRI and MRCP and a CE-MDCT. Invasive cholangiography should be reserved for histological diagnosis. In case of concomitant cholangitis or unresectable disease, therapeutic decompression via stent insertion is highly recommended. In cases of planned resection for hECC, the FLR should be drained to ensure appropriate postoperative liver regeneration [66].

### Definition of resectability

Whenever possible, patients with cholangiocarcinoma should be treated by surgery, representing the only treatment associated with long-term survival and possibility for cure. However, one third of patients present unresectable disease at time of diagnosis [66, 69]. Numerous studies reported that in order to achieve an R0 resection, an aggressive surgical approach (50–80% extended hepatectomy) is necessary [90, 91]. However, criteria for resectability are poorly defined, and vary considerably among institutions and countries. Most commonly accepted criteria defining non-resectability in cholangiocarcinoma are shown in Table 2. Besides patients’ fitness, from a technical and functional point of view, complete resectability first and foremost requires sufficient FLR volume in terms of healthy, vascularized parenchyma while ensuring appropriate biliary drainage options [92].

**Table 2:** Criteria defining unresectability in cholangiocarcinoma

Presence of distant metastases
Bilateral segmental ductal involvement
Extensive vascular involvement without ability for reconstruction
Unilateral atrophy with contralateral segmental duct or vascular inflow involvement
Unilateral segmental duct extension with contralateral vascular inflow involvement
*Specially for hilar CC:* Histologically involvement of N2 lymph nodes (celiac axis, superior mesenteric artery, pericaval, periaortic)

Several studies recommend a selective diagnostic laparoscopy to identify occult intraperitoneal or non-contiguous hepatic metastatic disease to avoid unnecessary laparotomy, especially in high-risk patients with a history of percutaneous stenting, large tumour size or markedly elevated serum CA19-9 levels. Staging laparoscopy identifies unexpected metastatic disease in 25–50% of patients, precluding the possibility for oncologically meaningful resection [93, 94].

### Type and extent of surgery

Tumour location (intrahepatic or along the biliary tract) and locoregional involvement determine the type and dimension of the operation. In case of ICC usually either AR or NAR is performed, with concomitant portal lymphadenectomy [66]. Treatment strategies for hECC vary according to tumour expansion along the bile duct reflected in the Bismuth–Corlette classification (Table 3). Extent of resection for Bismuth–Corlette type I hECCs depends on possible involvement of surrounding structures such as the portal vein, hepatic arteries or (caudate lobe) liver parenchyma and heavily relies on intraoperative frozen section analysis. Rigorous extension of the procedure (partial hepatectomy, vascular resection, pancreatic resection) to achieve tumour-free resection margins results in improved 5‑year OS of around 35% versus 8% after palliative resections [95]. Bismuth–Corlette type II–IV tumours usually require (extended) right or left hepatectomy with regional lymphadenectomy. Distal ECC necessitate radical pancreaticoduodenectomy for curative intent.

**Table 3:** Surgical treatment strategies for hECC according to the Bismuth–Corlette classification

Bismuth–Corlette type I tumours	Cholecystectomy Extrahepatic bile duct resection (±partial liver resection and vascular resections) Regional lymphadenectomy Bilioenteric anastomosis (Roux-Y hepaticojejunostomy)
Bismuth–Corlette type II, III tumours	Right or left hepatectomy with en bloc caudate lobectomy Regional lymphadenectomy
Bismuth–Corlette type IV tumours	Extended right or left hepatectomy with en bloc caudate lobectomy Regional Lymphadenectomy

Since tumour involvement of the caudate lobe is reported in over 50% of patients with hECC and segment 1 bile duct branches join the right and left hepatic ducts and/or their confluence, en bloc caudate resection is commonly recommended by most authors. A benefit in 5‑year OS of 25% after segment 1 resection versus 17.5% when compared to preservation has exemplarily been reported by Gazzaniga et al. [96].

Vascular involvement represents a major challenge in resection of cholangiocarcinoma. Several variants of liver resection combined with portal vein resection and reconstruction were reported, contributing to increased OS compared to non-resected patients [97]. Reconstruction is usually performed with end-to-end anastomosis or venous graft interposition, e. g. with the saphenous or external iliac vein. Although hepatic artery resection and reconstruction might be indicated in occasional cases of hECC, the resulting 5‑year OS of about 20% remains unsatisfactory and generates high rates of postoperative morbidity [98].

### Perioperative and survival outcomes

Postoperative morbidity is significant, with reported complication rates of 31–85% after cholangiocarcinoma resection, depending mainly on the type of resection (hECC > ICC). Specific complications include bile leakage, haemorrhage, PHLF, intraabdominal infection and portal vein thrombosis. Despite considerable improvements in surgical techniques and perioperative care, mortality after resection for cholangiocarcinoma still ranges from 3–17%, with PHLF being a major cause often combined with sepsis and multi-organ failure [91, 99].

Also, 5‑year survival rate following surgical resection currently remains between 30–35% for ICC [100] and 25–50% for hilar CC, with lymph node metastases limiting long-term survival [101]. Nevertheless, there is no evidence that extended lymph node dissection improves long-term survival [85]. Factors independently associated with improved survival are tumour-free resection margins, well-differentiated tumour grading, mass-forming type, absence of satellite nodules, lymph node involvement and vascular or perineural invasion, low CA-19-9 level and completion of adjuvant chemotherapy treatment [69, 100, 102–105]. Patients with microscopic tumour involvement with resection margins <1 mm (R1 resection) show limited survival, comparable to palliatively treated patients [99]. Even with complete resection, 50% of patients experience recurrence, with a mean time to recurrence of 10–20 months. Table 4 shows parameters associated with early recurrence [91, 94, 106].

**Table 4:** Parameters associated with early recurrence after resection of cholangiocarcinoma

Parameter associated with early recurrence
Tumour size >5 cm
Number of tumours
CA 19-9 levels >100 IU/ml
Vascular invasion
Tumour grading
Presence of lymph node metastases
Perineural invasion
Obstructive jaundice

Concerning adjuvant chemotherapy, most recently, results of the BILCAP study were presented at the 2017 Annual Meeting of the American Society of Clinical Oncology (ASCO). In this study comparing 6 months of capecitabine versus observation in over 400 patients, capecitabine was significantly associated with a 25% lower risk of death in the per protocol analysis [107]. Therefore, capecitabine should represent the standard of care in the adjuvant setting at present. Further combination regimens such as cisplatin-gemcitabine (ACTICCA-1 trial) are currently investigated in prospective, randomised studies [108].

### Liver transplantation

Data on the role of OLT for cholangiocarcinoma without preoperative treatment are limited so far (5-year survival rates 20–50%) compared to curative resection [94]. Studies showed improved 5‑year survival rates of over 70% when OLT was combined with neoadjuvant chemoradiotherapy in selected patients (the “Mayo protocol”) [66, 109, 110]. However, a multicentre analysis of the European Liver Transplant Registry in 2016 revealed comparably promising results without chemoradiation when the same strict selection criteria (hECC <3 cm, negative lymph nodes, no metastasis) were applied as in the Mayo study, therefore questioning the true contribution of pre-transplant therapy to overall survival [111]. A very recent multicentre study from the USA further challenged resection as the gold standard for hECC within these criteria, by showing that OLT (with preoperative treatment) results in substantially improved 5‑year OS of 54% versus 29% after resection [112].

### Palliative treatment

In contrast to the small group of patients presenting with resectable disease, the majority of cases show advanced disease at time of diagnosis. The benefit of palliative surgical resection is unclear. The aim of all palliative treatment options is to improve quality of life through relief of tumour-associated symptoms, e. g. from biliary or vascular obstruction [66].

### Biliary drainage

Most patients with advanced disease suffer from jaundice and require biliary drainage, which can be achieved either endoscopically or percutaneously, with a higher rate of successful placement and biliary decompression in the percutaneous technique [66]. Occlusion, migration and tumour ingrowth are the most commonly reported complications [110]. In palliative patients who have a life expectancy of more than 3–6 months, several guidelines recommend the use of metal stents over plastic stents due to fewer occlusion rates and less necessity for repeat ERCPs [66].

While the benefit of biliary stenting in palliative settings is proven, the beneficial effect of stenting prior to surgery is still unclear. A meta-analysis of four studies showed no significant differences in complication rates, morbidity and mortality between preoperative biliary stenting (PTC or endoscopic) and surgery alone in patients with obstructive jaundice [113]. As long as randomized controlled trials are missing, stenting prior to surgery is recommended in case of severe cholangitis or malnutrition and if extended liver resection is intended [66]. While there is consensus that for patients with an indication for stenting this should primarily be performed to clear the FLR side only [114], the applied technique (endoscopic vs. percutaneous) remains a matter of debate in daily practice. However, two meta-analyses have clearly shown increased post-interventional morbidity (OR 2.23) including cholangitis and pancreatitis, higher interventional conversion rates (to PTCD) and postoperative hepatic failure (after liver resection) in endoscopically stented patients [114, 115]. Nevertheless, evidence suggests, there may be an oncological long-term survival benefit of endoscopic biliary stenting due to less tumour seeding compared to PTCD [114, 116, 117].

## References

[1] Dünser M, Kwizera A (2017). Perioperative fluid management. Eur Surg.

[2] Poon RT, Fan ST, Lo CM, Liu CL, Lam CM, Yuen WK (2004). Improving perioperative outcome expands the role of hepatectomy in management of benign and malignant hepatobiliary diseases: analysis of 1222 consecutive patients from a prospective database. Ann Surg.

[3] Liau KH, Blumgart LH, DeMatteo RP (2004). Segment-oriented approach to liver resection. Surg Clin North Am.

[4] Gold JS, Are C, Kornprat P, Jarnagin WR, Gönen M, Fong Y (2008). Increased use of parenchymal-sparing surgery for bilateral liver metastases from colorectal cancer is associated with improved mortality without change in oncologic outcome: trends in treatment over time in 440 patients. Ann Surg.

[5] Torzilli G, Nagino M, Tzeng CD, Kingham TP, Alatise OI, Ayandipo OO (2017). SSAT State-of-the-Art Conference: New Frontiers in Liver Surgery. J Gastrointest Surg.

[6] Liver EAFTSOT, Cancer EOFRATO (2012). EASL-EORTC clinical practice guidelines: management of hepatocellular carcinoma. J Hepatol.

[7] Rous P, Larimore LD (1920). Relation of the portal blood to liver maintenance : a demonstration of liver atrophy conditional on compensation. J Exp Med.

[8] Jaeck D, Oussoultzoglou E, Rosso E, Greget M, Weber JC, Bachellier P (2004). A two-stage hepatectomy procedure combined with portal vein embolization to achieve curative resection for initially unresectable multiple and bilobar colorectal liver metastases. Ann Surg.

[9] Alvarez FA, Ardiles V, Sanchez Claria R, Pekolj J, de Santibanes E (2013). Associating liver partition and portal vein ligation for staged hepatectomy (ALPPS): tips and tricks. J Gastrointest Surg.

[10] Vennarecci G, Grazi GL, Sperduti I, Busi Rizzi E, Felli E, Antonini M (2016). ALPPS for primary and secondary liver tumors. Int J Surg.

[11] Schadde E, Ardiles V, Slankamenac K, Tschuor C, Sergeant G, Amacker N (2014). ALPPS offers a better chance of complete resection in patients with primarily unresectable liver tumors compared with conventional-staged hepatectomies: results of a multicenter analysis. World J Surg.

[12] Linecker M, Bjornsson B, Stavrou GA, Oldhafer KJ, Lurje G, Neumann U (2017). Risk adjustment in ALPPS is associated with a dramatic decrease in early mortality and morbidity. Ann Surg.

[13] Ulyett S, Shahtahmassebi G, Aroori S, Bowles MJ, Briggs CD, Wiggans MG (2017). Comparison of risk-scoring systems in the prediction of outcome after liver resection. Perioper Med (Lond).

[14] Aragon RJ, Solomon NL (2012). Techniques of hepatic resection. J Gastrointest Oncol.

[15] Imamura H, Seyama Y, Kokudo N, Maema A, Sugawara Y, Sano K (2003). One thousand fifty-six hepatectomies without mortality in 8 years. Arch Surg.

[16] Takayama T, Makuuchi M, Kubota K, Harihara Y, Hui AM, Sano K (2001). Randomized comparison of ultrasonic vs clamp transection of the liver. Arch Surg.

[17] Rau HG, Wichmann MW, Schinkel S, Buttler E, Pickelmann S, Schauer R (2001). Surgical techniques in hepatic resections: ultrasonic aspirator versus Jet-Cutter. A prospective randomized clinical trial. Zentralbl Chir.

[18] Ikeda M, Hasegawa K, Sano K, Imamura H, Beck Y, Sugawara Y (2009). The vessel sealing system (LigaSure) in hepatic resection: a randomized controlled trial. Ann Surg.

[19] Lesurtel M, Selzner M, Petrowsky H, McCormack L, Clavien PA (2005). How should transection of the liver be performed?: a prospective randomized study in 100 consecutive patients: comparing four different transection strategies. Ann Surg.

[20] Schwarz C, Klaus DA, Tudor B, Fleischmann E, Wekerle T, Roth G (2015). Transection Speed and Impact on Perioperative Inflammatory Response—A Randomized Controlled Trial Comparing Stapler Hepatectomy and CUSA Resection. PLoS ONE.

[21] Rahbari NN, Elbers H, Koch M, Vogler P, Striebel F, Bruckner T (2014). Randomized clinical trial of stapler versus clamp-crushing transection in elective liver resection. Br J Surg.

[22] Jemal A, Bray F, Center MM, Ferlay J, Ward E, Forman D (2011). Global cancer statistics. CA Cancer J. Clin..

[23] Venook AP, Papandreou C, Furuse J, de Guevara LL (2010). The incidence and epidemiology of hepatocellular carcinoma: a global and regional perspective. Oncologist.

[24] Spangenberg HC, Thimme R, Blum HE (2006). Serum markers of hepatocellular carcinoma. Semin Liver Dis.

[25] Witjes CD, ten Kate FJ, Verhoef C, de Man RA, IJzermans JN (2013). Immunohistochemical characteristics of hepatocellular carcinoma in non-cirrhotic livers. J Clin Pathol.

[26] Llovet JM, Bru C, Bruix J (1999). Prognosis of hepatocellular carcinoma: the BCLC staging classification. Semin Liver Dis.

[27] Finkenstedt A, Vikoler A, Portenkirchner M, Mulleder K, Maglione M, Margreiter C (2016). Excellent post-transplant survival in patients with intermediate stage hepatocellular carcinoma responding to neoadjuvant therapy. Liver Int.

[28] Sumie S, Nakashima O, Okuda K, Kuromatsu R, Kawaguchi A, Nakano M (2014). The significance of classifying microvascular invasion in patients with hepatocellular carcinoma. Ann Surg Oncol.

[29] Rodriguez-Peralvarez M, Luong TV, Andreana L, Meyer T, Dhillon AP, Burroughs AK (2013). A systematic review of microvascular invasion in hepatocellular carcinoma: diagnostic and prognostic variability. Ann Surg Oncol.

[30] Mazzaferro V, Regalia E, Doci R, Andreola S, Pulvirenti A, Bozzetti F (1996). Liver transplantation for the treatment of small hepatocellular carcinomas in patients with cirrhosis. N Engl J Med.

[31] Yao FY, Ferrell L, Bass NM, Watson JJ, Bacchetti P, Venook A (2001). Liver transplantation for hepatocellular carcinoma: expansion of the tumor size limits does not adversely impact survival. Hepatology.

[32] Mazzaferro V, Llovet JM, Miceli R, Bhoori S, Schiavo M, Mariani L (2009). Predicting survival after liver transplantation in patients with hepatocellular carcinoma beyond the Milan criteria: a retrospective, exploratory analysis. Lancet Oncol.

[33] Hong G, Suh KS, Suh SW, Yoo T, Kim H, Park MS (2016). Alpha-fetoprotein and (18)F-FDG positron emission tomography predict tumor recurrence better than Milan criteria in living donor liver transplantation. J Hepatol.

[34] Widmann G, Schullian P, Bale R (2013). Radiofrequency ablation of hepatocellular carcinoma. Wien Med Wochenschr.

[35] Gyori GP, Felsenreich DM, Silberhumer GR, Soliman T, Berlakovich GA (2017). Multimodality locoregional treatment strategies for bridging HCC patients before liver transplantation. Eur Surg.

[36] Zheng Z, Liang W, Milgrom DP, Zheng Z, Schroder PM, Kong NS (2014). Liver transplantation versus liver resection in the treatment of hepatocellular carcinoma: a meta-analysis of observational studies. Transplantation.

[37] Truty MJ, Vauthey JN (2010). Surgical resection of high-risk hepatocellular carcinoma: patient selection, preoperative considerations, and operative technique. Ann Surg Oncol.

[38] Imamura H, Sano K, Sugawara Y, Kokudo N, Makuuchi M (2005). Assessment of hepatic reserve for indication of hepatic resection: decision tree incorporating indocyanine green test. J Hepatobiliary Pancreat Surg.

[39] De Gasperi A, Mazza E, Prosperi M (2016). Indocyanine green kinetics to assess liver function: ready for a clinical dynamic assessment in major liver surgery?. World J Hepatol.

[40] Starlinger P, Pereyra D, Haegele S, Braeuer P, Oehlberger L, Primavesi F (2018). Perioperative von Willebrand factor dynamics are associated with liver regeneration and predict outcome after liver resection. Hepatology.

[41] Ogata S, Belghiti J, Farges O, Varma D, Sibert A, Vilgrain V (2006). Sequential arterial and portal vein embolizations before right hepatectomy in patients with cirrhosis and hepatocellular carcinoma. Br J Surg.

[42] Chan ACY, Chok K, Dai JWC, Lo CM (2017). Impact of split completeness on future liver remnant hypertrophy in associating liver partition and portal vein ligation for staged hepatectomy (ALPPS) in hepatocellular carcinoma: Complete-ALPPS versus partial-ALPPS. Surgery..

[43] Vennarecci G, Laurenzi A, Sandri LGB, Busi Rizzi E, Cristofaro M, Montalbano M (2014). The ALPPS procedure for hepatocellular carcinoma. Eur J Surg Oncol.

[44] Zhou Y, Xu D, Wu L, Li B (2011). Meta-analysis of anatomic resection versus nonanatomic resection for hepatocellular carcinoma. Langenbecks Arch Surg.

[45] Cucchetti A, Cescon M, Ercolani G, Bigonzi E, Torzilli G, Pinna AD (2012). A comprehensive meta-regression analysis on outcome of anatomic resection versus nonanatomic resection for hepatocellular carcinoma. Ann Surg Oncol.

[46] Tomimaru Y, Eguchi H, Marubashi S, Wada H, Kobayashi S, Tanemura M (2012). Equivalent outcomes after anatomical and non-anatomical resection of small hepatocellular carcinoma in patients with preserved liver function. Dig Dis Sci.

[47] Ryder SD, British Society of G (2003). Guidelines for the diagnosis and treatment of hepatocellular carcinoma (HCC) in adults. Gut.

[48] Elsayes KM, Hooker JC, Agrons MM, Kielar AZ, Tang A, Fowler KJ (2017). 2017 version of LI-RADS for CT and MR imaging: an update. Radiographics.

[49] Ishizawa T, Hasegawa K, Aoki T, Takahashi M, Inoue Y, Sano K (2008). Neither multiple tumors nor portal hypertension are surgical contraindications for hepatocellular carcinoma. Gastroenterology.

[50] Rajakannu M, Cherqui D, Ciacio O, Golse N, Pittau G, Allard MA (2017). Liver stiffness measurement by transient elastography predicts late posthepatectomy outcomes in patients undergoing resection for hepatocellular carcinoma. Surgery..

[51] Chong CC, Wong GL, Chan AW, Wong VW, Fong AK, Cheung YS (2017). Liver stiffness measurement predicts high-grade post-hepatectomy liver failure: A prospective cohort study. J Gastroenterol Hepatol.

[52] Lim C, Bhangui P, Salloum C, Gomez-Gavara C, Lahat E, Luciani A (2017). Impact of time to surgery in the outcome of patients with liver resection for BCLC 0‑A stage hepatocellular carcinoma. J Hepatol.

[53] Ciria R, Cherqui D, Geller DA, Briceno J, Wakabayashi G (2016). Comparative short-term benefits of laparoscopic liver resection: 9000 cases and climbing. Ann Surg.

[54] Komatsu S, Scatton O, Goumard C, Sepulveda A, Brustia R, Perdigao F (2017). Development process and technical aspects of laparoscopic hepatectomy: learning curve based on 15 years of experience. J Am Coll Surg.

[55] Ban D, Tanabe M, Ito H, Otsuka Y, Nitta H, Abe Y (2014). A novel difficulty scoring system for laparoscopic liver resection. J Hepatobiliary Pancreat Sci.

[56] Morise Z, Ciria R, Cherqui D, Chen KH, Belli G, Wakabayashi G (2015). Can we expand the indications for laparoscopic liver resection? A systematic review and meta-analysis of laparoscopic liver resection for patients with hepatocellular carcinoma and chronic liver disease. J Hepatobiliary Pancreat Sci.

[57] Fuks D, Cauchy F, Fteriche S, Nomi T, Schwarz L, Dokmak S (2016). Laparoscopy decreases pulmonary complications in patients undergoing major liver resection: a propensity score analysis. Ann Surg.

[58] Han HS, Shehta A, Ahn S, Yoon YS, Cho JY, Choi Y (2015). Laparoscopic versus open liver resection for hepatocellular carcinoma: case-matched study with propensity score matching. J Hepatol.

[59] Cheung TT, Dai WC, Tsang SH, Chan AC, Chok KS, Chan SC (2016). Pure laparoscopic hepatectomy versus open Hepatectomy for Hepatocellular carcinoma in 110 patients with liver cirrhosis: a propensity analysis at a single center. Ann Surg.

[60] Yoon YI, Kim KH, Kang SH, Kim WJ, Shin MH, Lee SK (2017). Pure laparoscopic versus open right Hepatectomy for Hepatocellular carcinoma in patients with cirrhosis: a propensity score matched analysis. Ann Surg.

[61] European Association For The Study Of The Liver, European Organisation For Research And Treatment Of Cancer (2012). EASL-EORTC clinical practice guidelines: management of hepatocellular carcinoma. J Hepatol.

[62] Bruix J, Takayama T, Mazzaferro V, Chau GY, Yang J, Kudo M (2015). Adjuvant sorafenib for hepatocellular carcinoma after resection or ablation (STORM): a phase 3, randomised, double-blind, placebo-controlled trial. Lancet Oncol.

[63] Zhuang L, Wen T, Xu M, Yang J, Wang W, Wu H (2017). Sorafenib combined with hepatectomy in patients with intermediate-stage and advanced hepatocellular carcinoma. Arch Med Sci.

[64] Jeng WJ, Lin CC, Chen WT, Sheen IS, Lin CY, Lin SM (2014). Adjuvant therapy for hepatocellular carcinoma after curative treatment. Dig Dis.

[65] Gao Z, Du G, Pang Y, Fu Z, Liu C, Liu Y (2017). Adjuvant transarterial chemoembolization after radical resection contributed to the outcomes of hepatocellular carcinoma patients with high-risk factors. Medicine (Baltimore).

[66] Khan SA, Davidson BR, Goldin RD, Heaton N, Karani J, Pereira SP (2012). Guidelines for the diagnosis and treatment of cholangiocarcinoma: an update. Gut.

[67] Bismuth H, Nakache R, Diamond T (1992). Management strategies in resection for hilar cholangiocarcinoma. Ann Surg.

[68] Deoliveira ML, Schulick RD, Nimura Y, Rosen C, Gores G, Neuhaus P (2011). New staging system and a registry for perihilar cholangiocarcinoma. Hepatology.

[69] Blechacz BR, Gores GJ (2008). Cholangiocarcinoma. Clin Liver Dis.

[70] Yamasaki S (2003). Intrahepatic cholangiocarcinoma: macroscopic type and stage classification. J Hepatobiliary Pancreat Surg.

[71] Olnes MJ, Erlich R (2004). A review and update on cholangiocarcinoma. Oncology.

[72] Cardinale V, Semeraro R, Torrice A, Gatto M, Napoli C, Bragazzi MC (2010). Intra-hepatic and extra-hepatic cholangiocarcinoma: New insight into epidemiology and risk factors. World J Gastrointest Oncol.

[73] Taylor-Robinson SD, Toledano MB, Arora S, Keegan TJ, Hargreaves S, Beck A (2001). Increase in mortality rates from intrahepatic cholangiocarcinoma in England and Wales 1968–1998. Gut.

[74] Khan SA, Taylor-Robinson SD, Toledano MB, Beck A, Elliott P, Thomas HC (2002). Changing international trends in mortality rates for liver, biliary and pancreatic tumours. J Hepatol.

[75] Shaib Y, El-Serag HB (2004). The epidemiology of cholangiocarcinoma. Semin Liver Dis.

[76] Patel T (2001). Increasing incidence and mortality of primary intrahepatic cholangiocarcinoma in the United States. Hepatology.

[77] Patel T (2002). Worldwide trends in mortality from biliary tract malignancies. BMC Cancer.

[78] West J, Wood H, Logan RF, Quinn M, Aithal GP (2006). Trends in the incidence of primary liver and biliary tract cancers in England and Wales 1971–2001. Br J Cancer.

[79] McGlynn KA, Tarone RE, El-Serag HB (2006). A comparison of trends in the incidence of hepatocellular carcinoma and intrahepatic cholangiocarcinoma in the United States. Cancer Epidemiol Biomarkers Prev.

[80] Shaib YH, Davila JA, McGlynn K, El-Serag HB (2004). Rising incidence of intrahepatic cholangiocarcinoma in the United States: a true increase?. J Hepatol.

[81] Khan SA, Toledano MB, Taylor-Robinson SD (2008). Epidemiology, risk factors, and pathogenesis of cholangiocarcinoma. HPB (Oxford).

[82] Welzel TM, McGlynn KA, Hsing AW, O’Brien TR, Pfeiffer RM (2006). Impact of classification of hilar cholangiocarcinomas (Klatskin tumors) on the incidence of intra- and extrahepatic cholangiocarcinoma in the United States. J Natl Cancer Inst.

[83] Lazaridis KN, Gores GJ (2005). Cholangiocarcinoma. Gastroenterology.

[84] McLean L, Patel T (2006). Racial and ethnic variations in the epidemiology of intrahepatic cholangiocarcinoma in the United States. Liver Int.

[85] Khan SA, Thomas HC, Davidson BR, Taylor-Robinson SD (2005). Cholangiocarcinoma. Lancet.

[86] Blechacz B, Komuta M, Roskams T, Gores GJ (2011). Clinical diagnosis and staging of cholangiocarcinoma. Nat Rev Gastroenterol Hepatol.

[87] Claessen MM, Vleggaar FP, Tytgat KM, Siersema PD, van Buuren HR (2009). High lifetime risk of cancer in primary sclerosing cholangitis. J Hepatol.

[88] Tyson GL, El-Serag HB (2011). Risk factors for cholangiocarcinoma. Hepatology.

[89] Bergquist A, von Seth E (2015). Epidemiology of cholangiocarcinoma. Best Pract Res Clin Gastroenterol.

[90] Farges O, Fuks D, Boleslawski E, Le Treut YP, Castaing D, Laurent A (2011). Influence of surgical margins on outcome in patients with intrahepatic cholangiocarcinoma: a multicenter study by the AFC-IHCC-2009 study group. Ann Surg.

[91] Endo I, Gonen M, Yopp AC, Dalal KM, Zhou Q, Klimstra D (2008). Intrahepatic cholangiocarcinoma: rising frequency, improved survival, and determinants of outcome after resection. Ann Surg.

[92] Mansour JC, Aloia TA, Crane CH, Heimbach JK, Nagino M, Vauthey JN (2015). Hilar cholangiocarcinoma: expert consensus statement. HPB (Oxford).

[93] Ruys AT, Busch OR, Gouma DJ, van Gulik TM (2011). Staging laparoscopy for hilar cholangiocarcinoma: is it still worthwhile?. Ann Surg Oncol.

[94] Simo KA, Halpin LE, McBrier NM, Hessey JA, Baker E, Ross S (2016). Multimodality treatment of intrahepatic cholangiocarcinoma: a review. J Surg Oncol.

[95] Zhao HL, Wei ZG, He JF, Liu JS, Zhao Y, Bao MS (2009). Experience of surgical resection of Bismuth-Corlette type I and II hilar cholangiocarcinoma. Zhonghua Wai Ke Za Zhi.

[96] Gazzaniga GM, Filauro M, Bagarolo C, Mori L (2000). Surgery for hilar cholangiocarcinoma: an Italian experience. J Hepatobiliary Pancreat Surg.

[97] Ebata T, Nagino M, Kamiya J, Uesaka K, Nagasaka T, Nimura Y (2003). Hepatectomy with portal vein resection for hilar cholangiocarcinoma: audit of 52 consecutive cases. Ann Surg.

[98] Matsuyama R, Mori R, Ota Y, Homma Y, Kumamoto T, Takeda K (2016). Significance of vascular resection and reconstruction in surgery for hilar cholangiocarcinoma: with special reference to hepatic arterial resection and reconstruction. Ann Surg Oncol.

[99] Esnaola NF, Meyer JE, Karachristos A, Maranki JL, Camp ER, Denlinger CS (2016). Evaluation and management of intrahepatic and extrahepatic cholangiocarcinoma. Cancer.

[100] de Jong MC, Nathan H, Sotiropoulos GC, Paul A, Alexandrescu S, Marques H (2011). Intrahepatic cholangiocarcinoma: an international multi-institutional analysis of prognostic factors and lymph node assessment. J Clin Oncol.

[101] Kobayashi A, Miwa S, Nakata T, Miyagawa S (2010). Disease recurrence patterns after R0 resection of hilar cholangiocarcinoma. Br J Surg.

[102] Sano T, Shimada K, Sakamoto Y, Yamamoto J, Yamasaki S, Kosuge T (2006). One hundred two consecutive hepatobiliary resections for perihilar cholangiocarcinoma with zero mortality. Ann Surg.

[103] Shaib YH, Davila JA, Henderson L, McGlynn KA, El-Serag HB (2007). Endoscopic and surgical therapy for intrahepatic cholangiocarcinoma in the united states: a population-based study. J Clin Gastroenterol.

[104] Ustundag Y, Bayraktar Y (2008). Cholangiocarcinoma: a compact review of the literature. World J Gastroenterol.

[105] Ramacciato G, Nigri G, Bellagamba R, Petrucciani N, Ravaioli M, Cescon M (2010). Univariate and multivariate analysis of prognostic factors in the surgical treatment of hilar cholangiocarcinoma. Am Surg.

[106] Hyder O, Hatzaras I, Sotiropoulos GC, Paul A, Alexandrescu S, Marques H (2013). Recurrence after operative management of intrahepatic cholangiocarcinoma. Surgery.

[107] Capecitabine Extends Survival for Biliary Tract Cancer. Cancer Discov. 2017;7(7):OF1. 10.1158/2159-8290.CD-NB2017-079 28572460

[108] Stein A, Arnold D, Bridgewater J, Goldstein D, Jensen LH, Klumpen HJ (2015). Adjuvant chemotherapy with gemcitabine and cisplatin compared to observation after curative intent resection of cholangiocarcinoma and muscle invasive gallbladder carcinoma (ACTICCA-1 trial)—a randomized, multidisciplinary, multinational phase III trial. BMC Cancer.

[109] Gu J, Bai J, Shi X, Zhou J, Qiu Y, Wu Y (2012). Efficacy and safety of liver transplantation in patients with cholangiocarcinoma: a systematic review and meta-analysis. Int J Cancer.

[110] Ebata T, Ercolani G, Alvaro D, Ribero D, Di Tommaso L, Valle JW (2016). Current status on cholangiocarcinoma and gallbladder cancer. Liver Cancer.

[111] Mantel HT, Westerkamp AC, Adam R, Bennet WF, Seehofer D, Settmacher U (2016). Strict selection alone of patients undergoing liver transplantation for hilar cholangiocarcinoma is associated with improved survival. PLoS ONE.

[112] Ethun CG, Lopez-Aguiar AG, Anderson DJ, Adams AB, Fields RC, Doyle MB (2018). Transplantation versus resection for hilar cholangiocarcinoma: an argument for shifting treatment paradigms for resectable disease. Ann Surg.

[113] Wang Q, Gurusamy KS, Lin H, Xie X, Wang C (2008). Preoperative biliary drainage for obstructive jaundice. Cochrane Database Syst Rev.

[114] Hameed A, Pang T, Chiou J, Pleass H, Lam V, Hollands M (2016). Percutaneous vs. endoscopic pre-operative biliary drainage in hilar cholangiocarcinoma—a systematic review and meta-analysis. HPB (Oxford).

[115] Al Mahjoub A, Menahem B, Fohlen A, Dupont B, Alves A, Launoy G (2017). Preoperative biliary drainage in patients with resectable perihilar cholangiocarcinoma: is percutaneous transhepatic biliary drainage safer and more effective than endoscopic biliary drainage? A meta-analysis. J Vasc Interv Radiol.

[116] Komaya K, Ebata T, Yokoyama Y, Igami T, Sugawara G, Mizuno T (2017). Verification of the oncologic inferiority of percutaneous biliary drainage to endoscopic drainage: A propensity score matching analysis of resectable perihilar cholangiocarcinoma. Surgery..

[117] Higuchi R, Yazawa T, Uemura S, Izumo W, Chaudhary RJ, Furukawa T (2017). ENBD is associated with decreased tumor dissemination compared to PTBD in perihilar cholangiocarcinoma. J Gastrointest Surg.

[118] Llovet JM, Burroughs A, Bruix J (2003). Hepatocellular carcinoma. Lancet.

